# Clinical and Prognostic Significance of Lupus Anticoagulant Measurement in Patients With Lung Cancer

**DOI:** 10.1177/1533034617714150

**Published:** 2017-06-12

**Authors:** Xianming Fei, Huan Wang, Lei Jiang, Tongwei Zhao, Maoliang Cheng, Wufeng Yuan

**Affiliations:** 1 Center of Laboratory Medicine, Zhejiang Provincial People’s Hospital, and People’s Hospital of Hangzhou Medical College, Hangzhou, China; 2 Department of Oncology, Zhejiang Provincial People’s Hospital, and People’s Hospital of Hangzhou Medical College, Hangzhou, China; 3 Department of Laboratory, Sir Run Run Hospital, Nanjing Medical University, Nanjing, China

**Keywords:** lupus anticoagulant, lung cancer, thrombotic complications, prognosis

## Abstract

Lupus anticoagulants is related to both recurrent thrombosis and cancer. Thrombotic complications occur more frequently in patients with lung cancer. The aim of this study is to investigate the association of lupus anticoagulants with hypercoagulability and thrombotic complications, as well as prognostic significance of lupus anticoagulants for patients with lung cancer. The study comprised 205 patients with non–small cell lung cancer. Plasma normalized LAC ratio, D-dimer, fibrinogen, activities of antithrombin, and FVIII before treatment were analyzed by coagulation analyzer, and routine hematologic and biochemical parameters were also evaluated. In patients, normalized LAC ratio, D-dimer, fibrinogen, and procoagulant activity of coagulating factor VIII levels significantly increased, whereas antithrombin activity significantly decreased compared with healthy controls (*P* < .001). Normalized LAC ratio was positively correlated with D-dimer, fibrinogen, and procoagulant activity of coagulating factor VIII, and negatively correlated with antithrombin activity, respectively (*P* < .01). D-dimer, procoagulant activity of coagulating factor VIII, and antithrombin levels revealed statistical difference in non–deep venous thrombosis patients with elevated or normal normalized LA ratio (*P* < .05). The incidence of deep venous thrombosis and tumor metastasis was higher, and 1-year survival rate was lower in elevated normalized LAC ratio patients than in normal ones, respectively (*P* < .01). There was higher normalized LAC ratio level in patients with deep venous thrombosis and/or metastasis (*P* < .05). In 1-year deceased patients, normalized LAC ratio level and the incidence of deep venous thrombosis and metastasis were higher than those in survivors, respectively (*P <* .05). Hazard regression analysis demonstrated normalized LAC ratio was independently associated with short survival time in patients with non–small cell lung cancer (hazard regression: 2.871, 95%confidence interval: 1.704-4.835; *χ^2^*: 19.130; *P* < .01). Our study suggests that lupus anticoagulants is a useful marker to predict thrombotic complications and prognosis in patient with lung cancer.

## Introduction

Antiphospholipid antibodies (APA) constitute a diverse group of autoantibodies reacting with phospholipids antigens. Antiphospholipid antibodies may be associated with a variety of diseases such as systemic lupus erythematosus (SLE), other autoimmune disorders, connective tissue disorders, malignancies, drug use, and infections or with no underlying disease.^1,2^ As a major APA, lupus anticoagulants (LAC) can increase in many diseases such as SLE, malignancies, infections, and so on.^3,4^ However, the actual mechanisms have not been fully elucidated. Lupus anticoagulants may increase the risk for thrombosis in patients without SLE and is defined as the risk factor for recurrent arterial and venous thromboembolism.^5,6^

Patients with tumors of lung, pancreas, and gastrointestinal tract are supposed to be more prone to hypercoagulable state, which increases the following disease progression.^7^ Disorders of coagulation function may greatly increase the overall risk for thrombotic complications, even lead to thrombotic events.^8,9^ Thrombosis is a frequent complication of malignant diseases, which is associated with short survival.^10^ Serious thrombotic complications such as venous thromboembolism (VTE), especially deep venous thrombosis (DVT), are the second leading causes of death in patients with cancer.^11,12^ As we know, patients with lung cancer usually exhibit more serious disorders of coagulation function, and higher risk for DVT than other malignancies patients, which would more easily result in metastasis of tumor cells, and recurrence of lung cancer.^13^ Therefore, patients with lung cancer would have higher morbidity and mortality.

It has been recognized that there was a higher prevalence of APA in patients with malignancies, and previous reports have indicated this association of APA with a large variety of malignancies.^3^ Patients at risk for thrombosis were characterized by the presence of LAC, and the thrombosis risk is connected solely with the presence of LAC.^14,15^ A retrospective study has showed that LAC was the risk factor of thrombosis in patients with lung cancer.^16^ Although LAC seems to be associated with thrombosis in patients with LAC-positive, the pathogenesis is actually unclear.^17^ The most popular hypotheses include the interference of LAC with protein C axis, disturbance of the annexin-A5 antithrombotic shield on the syncytiotrophoblast, interference of LAC with the complement system, and finally activation of many factors including coagulating factors, platelets, monocytes, and endothelial cells, which may lead to subsequent prothrombotic state and possible risk for thrombosis.^4^ On the other hand, tumor cells can activate coagulation system and lead to thrombosis mainly through the production of procoagulant, fibrinolytic, proaggregating activities, as well as release of proinflammatory and proangiogenic cytokines, and so on.^18^ Therefore, the abovementioned causes may all involve in the occurrence of hypercoagulable or prothrombotic state, even VTE in patients with cancer. Up to today, the significant association of LAC with cancer and thrombotic complications has been partly clarified.^3,4^ However, the roles of LAC in the hypercoagulable state and thrombosis are still unknown in patients with lung cancer, and there are not enough data to support whether LAC is really involved in the hypercoagulable state in patients with lung cancer and influences the coagulation function and prognosis of patients with lung cancer. Because NSCLC constitutes 85% of lung cancer,^13^ the aim of this study was to clarify the relationship between LAC and the hypercoagulable state, as well as the prognosis of patients with NSCLC, and also to understand the clinical importance of LAC in lung cancer.

## Materials and Methods

### Patients Population

A total of 205 patients with NSCLC, including 131 males and 74 females aged 43 to 79 [58.5 (28.5)] years from the Department of Oncology, Zhejiang Provincial People’s Hospital, China, between May 2013 and September 2014, were finally enrolled in our prospective cohort study. All patients were Han Chinese and diagnosed according to the histological diagnosis criteria, and there were 125 smokers in patients (95 males and 30 females). The pretreatment evaluation included the detailed clinical history and physical examination, the clinical data of patients during hospitalization, and the incidence of DVT formation and tumor metastasis at the time of diagnosis. Patients with DVT or tumor metastasis were screened through either typical or doubtful symptoms before treatment and were confirmed by subsequent compression ultrasonography or histopathology, respectively. The exclusion criteria were primary liver and kidney dysfunctions, postoperation, hypertension, cardiovascular and cerebrovascular diseases, complicating other malignancies, inflammation and infections, and other diseases potentially activating blood coagulation system, as well as antithrombotic agents-taking such as warfarin, heparin, aspirin, and traditional Chinese medicines influencing platelet and coagulating function in 2 weeks before samples collecting. And those patients who underwent chemotherapy in 8 weeks and radiation therapy or chemoradiation therapy in 4 weeks before the end of follow-up were not included in the study. According to the mutations measurements in advanced patients with NSCLC(≥III_b_), crizotinib and gefitinib were routinely used as the preferred drugs for those with echinoderm microtubule associated protein like 4-anaplastic lymphoma kinase (EML4-ALK) rearrangements and epithelial growth factor receptor (EGFR) mutations, respectively. However, postsurgical double-negative and nonadvanced patients (<III_b_) first received chemotherapy (taxol and cisplatin) combined with conventional conformal radiotherapy (3D-CRT) subsequently, and taxol and cisplatin were also used for the treatments of patients with mutations. For comparison, 102 healthy controls (60 smokers) matched for age, sex, and race were included in the analysis without taking any abovementioned antithrombotic agents in 2 weeks before samples collecting. Another 20 volunteers (10 males and 10 females, aged 20-66 years) participated in this study for normal plasma preparation. Informed consent was obtained from the patients, controls, and volunteers, and the study was authorized by the Hospital’s Ethics Committee.

### Laboratory Assays

Venous blood was collected from patients in the morning after patients fasting for 8 hours or more according to routine procedures before treatment. Blood was mixed with 3.8% trisodium citrate solution (9:1, vol/vol) and then centrifuged at 1500*g* for 10 minutes at room temperature to obtain platelet poor plasma (PPP). The normal plasma stored at −80^°^C for routine use in our laboratory was obtained from the 20 healthy volunteers before starting this study. An automatic coagulation analyzer and the commercially available LAC screening and confirmatory reagents (ACL TOP-700, Instrumentation Laboratory company, Lexington, MA, USA) were used for plasma normalized LAC ratio (NLR) measurements. In brief, coagulating time of normal plasma and PPP of patients and controls was measured with the screening and confirmatory reagents, respectively, and the screening time (ST) and confirmatory time (CT) were obtained. Subsequently, the screening ratio (SR) and confirmatory ratio (CR) were calculated (ST or CT of patients and controls divided by that of normal plasma), and NLR was also calculated according to the following formula:$\text{NLR}=\frac{\text{SR}}{\text{CR}}$. The calculating processes were performed automatically by the analyzer. Furthermore, another coagulation analyzer (Sysmex CS-5100, Japan) was used to measure the levels of D-dimer (D-D), antithrombin (AT) activity, fibrinogen (Fbg), procoagulant activity of coagulating factor ≤ (FVIII: C), prothrombin time (PT), and activated partial thromboplstin time (aPTT) using the matched reagents for hemostatic parameters (Siemens Healthcare Diagnostics Products GmbH, Marburg, Germany), respectively. And other routine hematologic and biochemical parameters including white blood cell (WBC) and platelet (PLT) counts, total protein (TP) and album (ALB), fasting serum glucose (GLU) were measured. Based on the measured results, it is thought to be elevated for NLR over or equal to 1.20 that was used as the cutoff value with clinical significance.

### Follow-Up Survey

For the posthospitalized survivor, a telephone follow-up was performed approximately every 1 month to investigate the survival and death information. Patients were followed prospectively either for 1 year, or until the occurrence of death. We calculated the total survival and mortality rate in 1 year of follow-up survey excluding the patients of accidental death. In this study, median follow-up time was 18.3 weeks (range: 5-52 weeks).

### Statistical Analysis

Student *t* test was used for 2 samples of continuous variables, and *χ^2^* test was used for categorical variables in the comparisons of patients with NSCLC with controls. The correlations between levels of NLR and other parameters were performed with Pearson correlation analysis. Survivors and deceased patients were determined using a Student *t* test where appropriate. Survival curve for the mortality of patients with NSCLC was drawn by Kaplan-Meier analysis according to the cut off value (1.20). Multivariate survival analysis was performed using Cox’s proportional hazards regression model, and log-rank test was used to test for survival between the groups. All statistical analysis were performed with SPSS software (version 17.0). *P* value of less than .05 was considered statistically significant.

## Results

The characteristics of the study population were presented in Table 1. Overall, about 70% were male, and the mean age at cancer diagnosis was 58.5 years old. In patients, male smokers constituted majority of the group (72.5%). Approximately 60% of the tumors were adenocarcinoma, and 30% were the type of squamous cell, and there was 21 (17.5%) of 120 patients with some mutations for adenocarcinoma, which included 17 (14.2%) with EGFR mutations, and 4 (3.3%) with EML4-ALK rearrangements (detailed data were not presented). At the time of diagnosis, 63.41% of patients had stage III_b_ or greater disease, and the incidence of DVT and tumor metastasis, and 1-year survival rate was 22.44%, 34.15%, and 70.73%, respectively. During the follow-up, 2 patients died from accidents.

**Table 1.** Characteristics of Patients With Non–Small Cell Lung Cancer.^a^

Characteristic	Number	Proportion, %
Sex (male)	141	68.78
Age (mean)	58.5	
NSCLC type		
Adenocarcinoma	120	58.54
Squamous cell	59	28.78
Others	26	12.68
Stage of NSCLC		
<III_b_	75	36.59
≥III_b_	130	63.41
Metastatic spread		
Yes	70	34.15
No	135	68.85
Complicating DVT		
Yes	46	22.44
No	159	77.56
1-year survival		
Yes	145	70.73
No	60	29.27

Abbreviations: DVT, deep venous thrombosis; NSCLC, non–small cell lung cancer.

^a^Data were presented as number and percentage.

**Figure 1.**
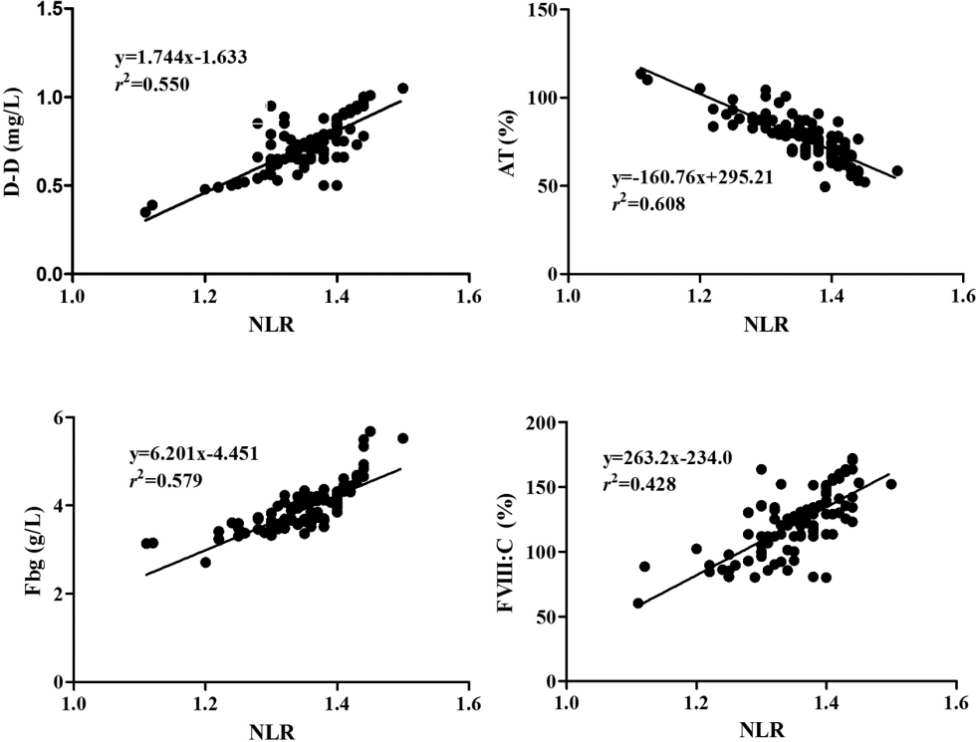
Correlation between NLR and levels of D-D, AT, Fbg, and FVIII: C in patients with NSCLC. AT indicates antithrombin; D-D, D-dimer, Fbg, fibrinogen; FVIII: C, procoagulant activity of coagulating factor VIII; NLR, normalized LAC ratio; NSCLC, non–small cell lung cancer.

Comparisons of NLR and other parameters levels in patients with NSCLC with those in controls were shown in Table 2. In this study, we obtained the coagulating time (ST: 31.3 s, CT: 30.2 s) of normal plasma. In the results, the mean age, percentage of male and smoking patients, WBC, PLT, body mass index, blood pressure, TP, ALB, GLU, PT, aPTT, CT, and CR levels did not reveal statistical difference between patients with NSCLC and controls (*P* > .05); whereas levels of ST, SR, NLR, D-D, Fbg, and FVIII: C increased significantly, and AT activities decreased in patients with NSCLC (*P* < .001). The correlations between NLR and hemostastic parameters were shown in Figure 1. Pearson correlation analysis indicated that NLR level was positively correlated with D-D, Fbg, and FVIII: C levels, respectively (*r =* 0.742, 0.761, and 0.65, *P* < .01), and negatively correlated with AT activity (*r =* −0.780, *P* < .01).

**Table 2.** Comparisons of NLR and Other Parameters of Patients With NSCLC With Controls.^a^

Variables	Patients With NSCLC	Controls	*P* Value
n	205	102	
Age, years	58.5 (15.1)	56.2 (14.0)	>.05
Sex (male/female)	128/77	62/40	>.05
BMI, kg/m^2^	26.3 (2.1)	25.6 (2.7)	>.05
Smoking patients (male/female)	95/30	43/17	>.05
SBP, mm Hg	131 (17)	130 (12)	>.05
DBP, mm Hg	81 (14)	78 (13)	>.05
Glucose, mmol/L	5.33 (1.51)	5.50 (1.72)	>.05
WBC, × 10^9^/L	6.18 (2.32)	5.12 (2.05)	>.05
PLT, × 10^9^/L	199 (22)	210 (24)	>.05
PT, s	11.3 (3.4)	11.7 (2.5)	>.05
aPTT, s	28.1 (5.0)	26.0 (3.8)	>.05
ST, s	37.6 (7.9)	31.4 (5.8)	<.01
CT, s	30.3 (5.3)	30.3 (5.1)	>.05
SR	1.23 (0.14)	1.03 (0.10)	<.01
CR	1.04 (0.09)	0.97 (0.10)	>.05
NLR	1.18 (0.15)	1.06 (0.06)	<.01
D-D, mg/L	0.79 (0.21)	0.56 (0.07)	<.01
AT, %	75.1 (12.0)	94.6 (11.2)	<.01
Fbg, g/L	3.99 (0.84)	3.01 (0.71)	<.01
FVIII:C, %	126.5 (19.4)	96.6 (14.1)	<.01

Abbreviations: AT, antithrombin; aPTT, activated partial thromboplstin time; BMI, body mass index; CR, confirmatory ratio; CT, confirmatory time; DBP, diastolic blood pressure; D-D, D-dimer; Fbg, fibrinogen; NSCLC, non–small cell lung cancer; NLR, normalized LAC ratio; PLT, platelet; PT, prothrombin time; SBP, systolic blood pressure; SD, standard deviation; SR, screening ratio; ST, screening time; WBC, white blood cell.

^a^Data were presented as mean (SD). *P* values are the compared results of NSCLC group with control group by Student *t* test.

Comparisons of hemostatic parameter levels in patients having NSCLC with different NLR levels were presented in Table 3. In patients without DVT, those with elevated NLR exhibited significantly higher levels of D-D and FVIII: C, and lower levels of AT activities than those with normal NLR (*P* < .05). However, there was no significant difference for Fbg levels between elevated and normal NLR groups (*P* > .05). And hemostatic parameters levels exhibit no statistical difference between patients with DVT with either normal or elevated NLR (*P* > .05).

**Table 3.** Comparisons of Hemostatic Parameters Levels in Patients Having NSCLC With Different NLR Levels.^a^

	Patients With DVT			Non-DVT Patients		
Parameters	Normal NLR	Elevated NLR	*P* Value	Normal NLR	Elevated NLR	*P* Value
n	17	29		104	55	
D-D, mg/L	0.85 (0.23)	0.90 (0.20)	.080	0.70 (0.23)	0.88 (0.26)	.016
AT %	70.2 (10.0)	66.3 (11.1)	.089	81.2 (9.5)	69.7 (10.3)	.006
Fbg,g/L	4.25 (0.77)	4.35 (0.70)	.125	3.88 (0.80)	3.93 (0.68)	.067
FVIII:C, %	130.0 (12.1)	135.1 (11.2)	.521	121.6 (13.2)	130.2 (12.0)	.035

Abbreviations: AT, antithrombin; D-D, D-dimer; DVT, deep venous thrombosis; Fbg, fibrinogen; NLR, normalized LAC ratio; SD, standard deviation.

^a^Data were presented as mean (SD). *P* values are the compared results of normal NLR group with elevated NLR group by Student *t* test.

**Figure 2.**
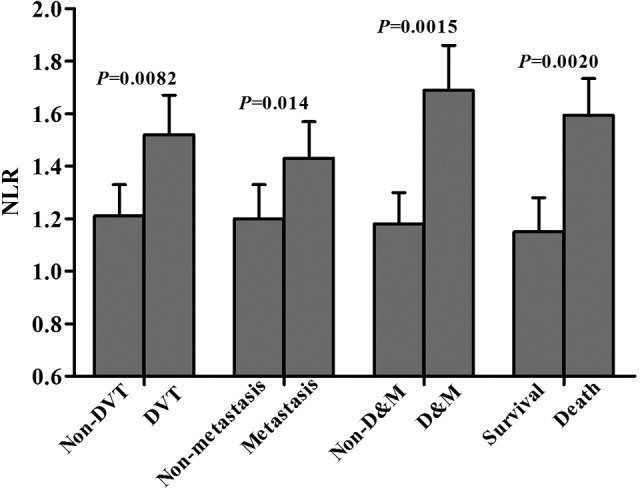
Comparisons of NLR in different clinical outcomes of patients with NSCLC. D&M, complicating both DVT and metastasis. DVT indicates deep venous thrombosis; NLR, normalized LAC ratio; NSCLC, non–small cell lung cancer.

Association of NLR and histological type with the incidence of DVT, tumor metastasis, and 1-year survival in patients with NSCLC was shown in Table 4. Compared with normal NLR patients, those with elevated NLR have higher incidence of DVT and tumor metastasis, and lower 1-year survival rate (*P* < .01), whereas the incidence of DVT, tumor metastasis, and 1-year survival did not exhibit significant difference between patients with adenocarcinoma and other histological types (*P* > .05). The difference of NLR levels in patients having NSCLC with different clinical outcomes was presented in Figure 2. In patients having NSCLC with DVT, metastasis, both DVT and metastasis, or 1-year deceased, levels of NLR were significantly increased when compared with those without the abovementioned clinical outcomes, respectively (*P* < .05). Comparisons of the incidence of DVT and tumor metastasis between 1-year survivors and deceased patients were presented in Table 5. In 1-year survivors, the incidence of DVT and tumor metastasis was significantly lower than that in deceased patients (*P* < .001).

**Table 4.** Comparisons of NLR and Histology Type With the Incidence of DVT, Tumor Metastasis, and 1-Year Survival in Patients With NSCLC.^a^

	NLR			Histology Type		
Clinical Outcomes	Normal Group	Elevated Group	*P* Value	Adenocarcinoma	Other Types	*P* Value
n	121	84		120	85	
DVT, n (%)	17 (14.0)	29 (34.5)	0.00	25 (20.83)	21 (24.71)	>.05
Metastasis, n (%)	27 (22.3)	43 (51.2)	0.00	44 (36.67)	26 (30.59)	>.05
Survival, n (%)	97 (80.2)	48 (57.1)	0.00	82 (68.33)	63 (74.12)	>.05

Abbreviations: DVT, deep venous thrombosis; NLR, normalized LAC ratio.

^a^Data were presented as numbers (percentage). *P* values are the compared results of normal NLR with elevated NLR as well as adenocarcinoma with other types by χ^2^ test.

**Table 5.** Comparisons of the Incidence of DVT and Metastasis in 1-Year Survivors and Deceased Patients.^a^

	One-Year Survivors	Deceased Patients	*P* Value
n	145	60	
DVT, n (%)	10 (6.9)	36 (60.0)	.00
Metastasis, n (%)	16 (11.0)	54 (90.0)	.00

Abbreviation: DVT, deep venous thrombosis.

^a^Data were presented as numbers (percentage). *P* values are the compared results of 1-year survivors and deceased patients.

**Figure 3.**
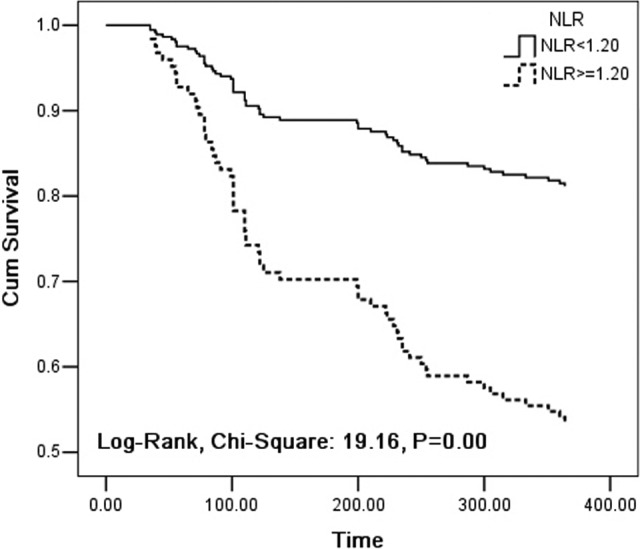
Overall survival curves according to NLR levels in patients with NSCLC. *P* = .00: Comparison of NLR <1.20 (real line) with NLR ≥1.20 (broken line). NLR indicates normalized LAC ratio; NSCLC, non–small cell lung cancer.

Results of survival analysis were presented in Table 6 and Figure 3. Hazard regression analysis revealed that there was not significant impact of biologic variables including age, gender, and smoking status on survival (*P* > .05), whereas NLR was independently associated with worse survival (HR: 2.871, 95%CI: 1.704-4.835, *P <* .001). And log-rank test showed that patients with elevated NLR (≥1.20) had shorter overall survival time than that with normal NLR (<1.20; mean survival time: [244.3 ± 15.3] days vs [343.6 ± 4.5] days, *P <* .001).

**Table 6.** Results of Cox’s Proportional Hazard Regression Analysis in Patients With NSCLC.

Variables	Relative Risk	95% CI	*P* Value
Age	0.786	0.514-1.202	>.05
Gender	1.677	0.955-2.948	>.05
Smoking	1.002	0.970-1.034	>.05
NLR	2.871	1.704-4.835	<.001

Abbreviations: CI, confidence interval; NLR, normalized LAC ratio.

## Discussion

Many studies demonstrated that there was higher incidence of DVT and metastatic spread, and mortality in patients with lung cancer.^18,19^ In the present study, both male individuals and male smokers constituted majority of the groups, which indicated that there might be higher incidence of lung cancer in male smoking individuals. At the time of diagnosis, 63.41% of patients had stage III_b_ or greater disease, and the incidence of DVT, metastatic spread, and 1-year mortality was 22.44%, 34.15%, and 29.27% in patients with NSCLC. The incidence of DVT in our study was higher than those about 11% and 1% to 8% reported by other studies.^15,19^ The difference may result from different population race, verification of venous thrombosis, and longer courses of disease before diagnosis of the patients in this study.

Lupus anticoagulants may exhibit different levels in patients with malignancies, and high frequency of LAC was confirmed in patients with lung cancer.^3,16^ Some studies have shown that disorder of coagulating function may cause an initial hypercoagulable state with subclinical abnormalities and lead to abnormal levels of some hemostatic markers such as D-D, Fbg, FVIII, and AT in patients with cancer.^20–23^ In the present study, we found significantly increased levels of D-D, Fbg, and FVIII: C, and decreased AT activity in patients with NSCLC. The results indicated that patients with hypercoagulable state may be at greater risk in developing thrombotic complications than those without such disorders. Furthermore, we also found that NLR was markedly elevated in patients with NSCLC, while PT and aPTT did not reveal statistical difference between the 2 groups, which could be explained by what the prolongating effect of LA on aPTT compensated by coagulating factors activation in some patients. At the same time, we further observed that NLR was positively correlated with the levels of D-D, Fbg, and FVIII: C and negatively with AT activities. All the abovementioned results revealed that LAC-positive was in accordance with abnormal levels of hemostatic parameters in patients with NSCLC, and it might be a useful marker indicating coagulation activation in patients with lung cancer. Another important finding was that there were more abnormal levels of hemostatic parameters in non-DVT patients with elevated NLR than those with normal NLR, which also further revealed that elevated NLR probably accompanied abnormal coagulating, fibrinolytic, and anticoagulant ability, and might be associated with hypercoagulable state to some extend in patients with NSCLC. Therefore, our findings strongly suggest that LAC may be closely correlated with disorders of hemostatic/anticoagulatin/fibrinolytic systems and play important roles in the progression of hypercoagulable state and venous thrombotic complications in patients with NSCLC. As we know, besides LAC, other procoagulants from tumor cell can increase the risk for VTE, and it would be more necessary to receive some antithrombotic drugs for VTE prophylaxis and prognostic improvement in patients having NSCLC with LAC-positive, which would be the most important to prevent patients from thrombotic complications for patients having lung cancer with positive LAC.

Lung cancer is a seriously life-threatening malignancy. Patients with lung cancer have higher mortality, and numerous causes are involved in the death of patients.^8^ It has been recognized that VTE is the second leading cause of death in patients with cancer, and lots of studies have provided the evidences that VTE is closely related to tumor metastasis and worse prognosis.^17^ Specially, patients having cancer with thrombosis have a 4- to 6-fold higher risk of dying after an acute thrombotic event than those patients without cancer.^24^ Furthermore, patients with cancer and thrombosis have a 2- to 3-fold lower survival rate than those with cancer and without thrombosis.^25^ Therefore, it is important to prevent patients with lung cancer from thrombotic complications and to decrease mortality rates. In the present study, we found that patients with elevated NLR had higher incidence of DVT and tumor metastasis and lower 1-year survival rate than patients with normal NLR. The finding suggested that LAC-positive was associated with the occurrence of DVT and tumor metastasis and might increase the probability of death in patients with NSCLC. Another finding was that patients having NSCLC with different histology types did not exhibit markedly different incidence of DVT and tumor metastasis, as well as 1-year survival rate, which seemed to reveal that histology type was not correlated with the occurrence of DVT and tumor metastasis, and also did not significantly influence survival time of patients with NSCLC. And our study also further showed that NLR was significantly increased in patients with DVT or/and metastasis, and higher NLR level in 1-year deceased patients than in survivors, which revealed that patients with thrombotic complications or/and metastatic spread, as well as short survival time, might have much higher NLR levels. As expected, our finding also showed that the incidence of DVT and tumor metastasis in 1-year deceased patients was significantly higher than that in survivors. Although there was no evidences that LAC directly leaded to decreased survival time, the study also further indicated that there might be a tendency toward decreased survival time for patients having NSCLC with elevated NLR, and LAC-positive was associated with short survival time to some extent in patients with lung cancer. Therefore, LAC can be used as a valuable predicting marker for thrombotic complications, metastasis, and prognosis of patient with lung cancer patient.

High levels of circulating hemostatic markers have been revealed the prognostic value which was associated with decreased survival time for lung cancer.^7,26^ Our studies have also revealed the significant association of LAC with disorders of coagulation function and decreased survival time. To confirm the prognostic value of LAC-positive in patients with NSCLC, we performed the multivariate survival analysis. We found that there was no obvious effect of biologic parameters on survival time in patients with NSCLC, whereas elevated NLR was strongly associated with worse survival (HR: 2.871), and patients with elevated NLR (≥1.20) had significantly shorter survival time (244.3 ± 15.3 days) compared with those with normal NLR (<1.20; 343.6 ± 4.5 days), which further revealed that elevated NLR might be the risk factor for short survival time of patient with NSCLC and suggested that LAC-positive was probably associated with worse prognosis in patient with lung cancer. For patients with cancer, although thrombotic complications are the important factors causing patients to die, there are some other risk factors. As we know, besides LAC, other procoagulants from tumor cell can increase the risk for VTE, and metastatic spread would exacerbate the process of VTE development. Therefore, it is important to measure LAC and other hemostatic markers to assist oncologist in risk evaluation of hypercoagulable state and thrombotic complications in patients with cancer. Moreover, it would be more necessary to receive some antithrombotic drugs for VTE prophylaxis and prognostic improvement for patients having NSCLC with LAC-positive.

In this study, there may be several limitations. First, direct markers of coagulation activation are important to assess the relationship between LAC and disorders of coagulation function. Although we did not measure them, we have found that LAC-positive was significantly correlated with the indirect markers and closely associated with DVT and tumor metastasis, as well as short survival time, which revealed its clinical significance in patients with lung cancer. Second, only symptomatic or doubtful DVT or metastasis patients were included, and we cannot exclude that asymptomatic DVT or metastasis which might be present in some patients and potentially decreased the predicting power of LAC-positive for them. However, our study still demonstrated its important predicting values for DVT, tumor metastasis, and survival. Third, there probably were some patients having NSCLC with confounding factors such as disease status at the time of diagnosis, different therapeutic schedules, types of drug, and responses to therapy, and these factors would potentially influence tumor metastasis and prognosis, and decreased the predicting power of LAC. However, based on the significant correlation between LAC and DVT, our study also revealed the significant effect of LAC on tumor metastasis and prognosis of patients with NSCLC.

In conclusion, our study suggests that LAC is significantly correlated with venous thrombotic complications in patients with NSCLC, and there probably will be worse prognosis for LAC-positive patients with lung cancer. It may provide a foresight about the coagulating abnormalities and outcomes by using LAC measurements in patients with lung cancer. However, further controlled prospective studies on large groups of patients and longer time of follow-up may give more definite results and further help to stratify patients based on the presence of LAC in patients with lung cancer.

## Abbreviations

ALB: album
APA: antiphospholipid antibodies
aPTT: activated partial thromboplstin time
AT: antithrombin
CI: confidence interval
CR: confirmatory ratio
CT: confirmatory time
D-D: D-dimer
DVT: deep venous thrombosis
Fbg: fibrinogen
FVIII: C: procoagulant activity of coagulating factor VIII
GLU: fasting serum glucose
HR: hazard regression
LAC: lupus anticoagulants
NSCLC: non–small cell lung cancer
NLR: plasma normalized LAC ratio
PLT: platelet
PPP: platelet poor plasma
PT: prothrombin time
SLE: systemic lupus erythematosus
SR: screening ratio
ST: screening time
TP: total protein
VTE: venous thromboembolism
WBC: white blood cell

## References

[1] Rosborough TK Jacobsen JM Shepherd MF Factor X and factor II activity levels do not always agree warfarin-treated lupus anticoagulant patients. Blood Coagul Fibrinolysis. 2010;21(3):242–244. 20182349 10.1097/MBC.0b013e32833581a3

[2] Peker E Kavakli K Balkan C Karapinar D Aydemir B Incidence and clinical importance of lupus anticoagulant in children with recurrent upper respiratory tract Infection. Clin App Thromb Hemost. 2011;17(2):220–224. 10.1177/1076029609351292 19903696

[3] Tincani A Taraborelli M Cattaneo R Antiphospholipid antibodies and malignancies. Autoimmun Rev. 2010;9(4):200–202. 19386286 10.1016/j.autrev.2009.04.001

[4] Musiał J Antiphospholipid antibodies and thrombosis. Thromb Res. 2012;129(3):345–347. 22119156 10.1016/j.thromres.2011.10.029

[5] Gebhart J Lechner K Skrabs C Lupus anticoagulant and thrombosis in splenic marginal zone lymphoma. Thrombo Res. 2014;134(5):980–984. 10.1016/j.thromres.2014.08.021 25201005

[6] Reynaud Q Lega JC Mismetti P Risk of venous and arterial thrombosis according to type of antiphospholipid antibodies in adults without systemic lupus erythematosus: a systematic review and meta-analysis. Autoimmun Rev. 2014;13(6):595–608. 24418303 10.1016/j.autrev.2013.11.004

[7] Tas F Kilic L Serilmez M Keskin S Sen F Duranyildiz D Clinical and prognostic significance of coagulation assays in lung cancer. Respir Med. 2013;107(3):451–457. 23200643 10.1016/j.rmed.2012.11.007

[8] Radhakrishna G Berridge D Cancer-related venous thromboembolic disease: current management and areas of uncertainty. Phlebology. 2012;27(suppl 2):53–60. 22457305 10.1258/phleb.2012.012s34

[9] Khorana AA Connolly GC Assessing risk of venous thromboembolism in the patient with cancer. J Clin Oncol. 2009;27(29):4839–4847. 19720906 10.1200/JCO.2009.22.3271 PMC2764392

[10] Ogino H Hayashi S Kawasaki M Nakanishi M Hara N Association of thrombosis-inducing activity (TIA) with fatal hypercoagulable complications in patients with lung cancer. ChesChest. 1994;105(6):1683–1686. 10.1378/chest.105.6.1683 8205861

[11] Stricker H Venous thromboembolism and cancer: pathophysiology and incidence. Vasa. 2014;43(4):239–243. 25007901 10.1024/0301-1526/a000358

[12] Elyamany G Alzahrani AM Bukhary E Cancer-associated thrombosis: an overview. Clin Med Insights Onco. 2014;8:129–137. 10.4137/CMO.S18991 PMC4259501 25520567

[13] Galli M Luciani D Bertolini G Barbui T Lupus anticoagulants are stronger risk factors for thrombosis than anticardiolipin antibodies in the antiphospholipid syndrome: a systematic review of the literature. Blood. 2003;101(5):1827–1832. 12393574 10.1182/blood-2002-02-0441

[14] Ruffatti A Del Ross T Ciprian M Risk factors for a first thrombotic event in antiphospholipid antibody carriers: a prospective multicentre follow-up study. Ann Rheum Dis 2011;70(6):1083–1086. 21285115 10.1136/ard.2010.142042

[15] De Meis E Pinheiro VR Loures MA Lupus anticoagulant activity as a thrombosis risk factor in lung adenocarcinoma patient. Ann N Y Acad Sci. 2007;1107:51–55. 17804532 10.1196/annals.1381.006

[16] Tripodi A de Groot PG Pengo V Antiphospholipid syndrome: laboratory detection, mechanisms of action and treatment. J Intern Med. 2011;270(2):110–122. 21323768 10.1111/j.1365-2796.2011.02362.x

[17] Noble S Pasi J Epidemiology and pathophysiology of cancer-associated thrombosis. Br J Cancer. 2010;102(suppl 1):S2–S9. 20386546 10.1038/sj.bjc.6605599 PMC3315367

[18] Tagalakis V Levi D Agulnik JS Cohen V Kasymjanova G Small D High risk of deep vein thrombosis in patients with non-small cell lung cancer: a cohort study of 493 patients. J Thorac Oncol 2007;2(8):729–734. 17762339 10.1097/JTO.0b013e31811ea275

[19] Königsbrügge O Pabinger I Ay C Risk factors for venous thromboembolism in cancer: novel findings from the Vienna Cancer and Thrombosis Study (CATS). Thromb Res. 2014;133(suppl 2):S39–S43 24862144 10.1016/S0049-3848(14)50007-2

[20] Franchini M Montagnana M Targher G Manzato F Lippi G Pathogenesis, clinical and laboratory aspects of thrombosis in cancer. J Thromb Thrombolysis. 2007;24(1):29–38. 17396228 10.1007/s11239-007-0028-6

[21] Ay C Dunkler D Pirker R High d-dimer levels are associated with poor prognosis in cancer patients. Haematologia. 2012;97(8):1158–1164. 10.3324/haematol.2011.054718 PMC3409812 22371182

[22] Farrell DH γ′ Fibrinogen as a novel marker of thrombotic disease. Clin Chem Lab Med. 2012;50(11):1903–1909. 23091268 10.1515/cclm-2012-0005

[23] Payne AB Miller CH Hooper WC Lally C Austin HD High factor VIII, von Willebrand factor, and fibrinogen levels and risk of venous thromboembolism in blacks and whites. Ethn Dis. 2014;24(2):169–174. 24804362 PMC4618385

[24] Prandoni P Bernardi E Marchiori A The long-term clinical course of acute deep vein thrombosis of the arm: prospective cohort study. BMJ. 2004;329(7464):484–485. 15256419 10.1136/bmj.38167.684444.3A PMC515197

[25] Sørensen HT Mellemkjaer L Olsen JH Baron JA Prognosis of cancer associated with venous thromboembolism. N Engl J Med. 2000;343(25):1846–1850. 11117976 10.1056/NEJM200012213432504

[26] Buccheri G Ferrigno D Ginardi C Zuliani C Haemostatic abnormalities in lung cancer: prognostic implications. Eur J Cancer. 1997;33(1):50–55. 9071899 10.1016/s0959-8049(96)00310-3

